# Histopathological Correlations of Qualitative and Quantitative Temporopolar MRI Analyses in Patients With Hippocampal Sclerosis

**DOI:** 10.3389/fneur.2021.801195

**Published:** 2021-12-24

**Authors:** Bruna Cunha Zaidan, Ingrid Carolina da Silva Cardoso, Brunno Machado de Campos, Luciana Ramalho Pimentel da Silva, Vanessa C. Mendes Coelho, Kairo Alexandre Alves Silveira, Bárbara Juarez Amorim, Marina Koutsodontis Machado Alvim, Helder Tedeschi, Clarissa Lin Yasuda, Enrico Ghizoni, Fernando Cendes, Fabio Rogerio

**Affiliations:** ^1^Department of Pathology, School of Medical Sciences, University of Campinas (UNICAMP), Campinas, Brazil; ^2^Department of Neurology, School of Medical Sciences, University of Campinas (UNICAMP), Campinas, Brazil; ^3^Department of Anesthesiology, Oncology and Radiology, School of Medical Sciences, University of Campinas (UNICAMP), Campinas, Brazil

**Keywords:** epilepsy, hippocampal sclerosis, white matter, immunohistochemistry, MRI, diffusion weighted imaging

## Abstract

Hippocampal sclerosis (HS) is a common cause of pharmacoresistant focal epilepsy. Here, we (1) performed a histological approach to the anterior temporal pole of patients with HS to evaluate cortical and white matter (WM) cell populations, alteration of myelin integrity and markers of neuronal activity, and (2) correlated microscopic data with magnetic resonance imaging (MRI) findings. Our aim was to contribute with the understanding of neuroimaging and pathophysiological mechanisms of temporal lobe epilepsy (TLE) associated with HS. We examined MRIs and surgical specimens from the anterior temporal pole from TLE-HS patients (*n* = 9) and compared them with 10 autopsy controls. MRIs from healthy volunteers (*n* = 13) were used as neuroimaging controls. Histological techniques were performed to assess oligodendrocytes, heterotopic neurons, cellular proliferative index, and myeloarchitecture integrity of the WM, as well as markers of acute (c-fos) and chronic (ΔFosB) activities of neocortical neurons. Microscopic data were compared with neuroimaging findings, including T2-weighted/FLAIR MRI temporopolar blurring and values of fractional anisotropy (FA) from diffusion-weighed imaging (DWI). We found a significant increase in WM oligodendrocyte number, both in hematoxylin and eosin, and in Olig2-stained sections. The frequencies of oligodendrocytes in perivascular spaces and around heterotopic neurons were significantly higher in patients with TLE–HS compared with controls. The percentage of 2',3'-cyclic-nucleotide 3'-phosphodiesterase (CNPase; a marker of myeloarchitecture integrity) immunopositive area in the WM was significantly higher in TLE-HS, as well as the numbers of c-fos- and ΔFosB-immunostained neocortical neurons. Additionally, we demonstrated a decrease in axonal bundle integrity on neuroimaging, with a significant reduction in the FA in the anterior temporal pole. No differences were detected between individuals with and without temporopolar blurring on visual MRI analysis, considering the number of oligodendroglial cells and percentage of WM CNPase-positive areas. Also, there was no relationship between T2 relaxometry and oligodendrocyte count. In conclusion, our histopathological data support the following: (1) the hypothesis that repetitive neocortical neuronal activity could induce changes in the WM cellular constitution and myelin remodeling in the anterior temporal pole from patients with TLE-HS, (2) that oligodendroglial hyperplasia is not related to temporal blurring or T2 signal intensity on MRI, and (3) that reduced FA is a marker of increase in Olig2-immunopositive cells in superficial temporopolar WM from patients with TLE-HS.

## Introduction

Epilepsy is one of the most common neurological diseases and may be associated with several conditions leading to recurrent epileptic seizures with different presentation patterns (1). In most patients, pharmacological treatment is effective for controlling seizures. However, 20–30% of individuals do not respond well to antiseizure medications and, therefore, are potential candidates for surgical procedures (2, 3). According to the consensus by the International League Against Epilepsy (ILAE), pharmacoresistant epilepsy can be defined as the failure to remit seizures with two adequate regimens of antiseizure medication in either combination or monotherapy (4).

Among the different pathological conditions that cause focal epilepsy, hippocampal sclerosis (HS) is the most frequent histopathological diagnosis in surgical specimens of pharmacoresistant temporal lobe epilepsy (TLE) in adults (5). The pharmacoresistance rate in epilepsy varies depending on the etiology. Specifically, in patients with HS such rate ranges from 75.3 to 90% (6, 7). Morphological changes in the white matter (WM), including oligodendroglial hyperplasia and increased numbers of heterotopic neurons, have been described for decades in TLE surgical specimens (8–10). Additionally, recent experimental studies support the idea that repetitive neuronal activity may induce changes in cell constitution and myelin remodeling in the WM (11), suggesting a maladaptive response.

There are few studies on the correlation of neuroimaging findings with WM microscopic abnormalities in patients with epilepsy. Garbelli et al. (12) performed T2-weighted 7T magnetic resonance imaging (MRI), histological and ultrastructural protocols to investigate surgical samples from individuals with TLE associated with HS and loss of the delimitation between the gray and white matter of the temporal pole on MRI. The authors concluded that such MRI temporopolar blurring on T2-weighted and fluid attenuated inversion recovery (FLAIR) images would be a macroscopic presentation of a long-lasting axonal degeneration and redistribution of the remaining fibers.

In this work, we performed a histopathological, immunohistochemical and neuroimaging approach to the anterior pole of the temporal lobe of patients with HS, with and without MRI T2-weighted/FLAIR temporopolar blurring. These individuals were chosen because HS is a frequent cause of pharmacoresistant focal epilepsy, and no structural change was detected in the neocortical region adjacent to the WM under investigation. Our aim was to study WM changes and associate them with expression of markers of acute and chronic activity in neocortical neurons, thus contributing with the understanding of neuroimaging data (including MRI T2-weighted/FLAIR temporopolar blurring) and with findings supporting pathophysiological mechanisms associated with neuronal activation and WM changes in human TLE-HS specimens.

## Materials and Methods

This study was approved by the Research Ethics Committee of our Institution (#3.087.964) and consisted of retrospective analyses of tissue samples and neuroimaging data from individuals with HS and controls.

Specifically, the cohort of patients with HS (*n* = 9) was the same for both histopathological and MRI evaluations. As an inclusion criteria, we enrolled patients (i) from 2013 to 2018 with clinical diagnosis of pharmacoresistant TLE, MRI signs of HS with and without T2-weighted/FLAIR temporopolar blurring, and a comprehensive preoperative investigation in our tertiary epilepsy center, including EEG, neuroimaging (3 T MRI acquisition, using an 8-channel standard head coil) and neuropsychological examination; (ii) submitted to a temporopolar amygdalohippocampectomy, which led to an en bloc resection of the hippocampus and removal of the anterior temporal pole [according to the formerly published techniques (13)]; (iii) whose surgical histopathological report confirmed the diagnosis of HS [all of them were classified as type I according to the 2013 ILAE proposal (14)] and, (iv) whose paraffin blocks containing the anterior temporal pole specimen were adequately preserved and with tissue amount sufficient to allow for immunohistochemical reactions. Patients who did not fulfill one of the above-mentioned criteria were excluded from the study.

For histopathological evaluation, the control group consisted of adults submitted to autopsy (*n* = 10) with neither neurological disease nor history of seizures. Their anterior temporal pole was sampled as part of a local protocol for central nervous system postmortem evaluation. All autopsy tissues were collected between 6 and 12 h after death. To avoid interference with the microscopic assessment, only samples in adequately preserved paraffin blocks and that did not show signs of autolysis (eosinophilic cytoplasm and/or pyknotic nuclei) were selected.

For MRI acquisition, the control group consisted of healthy controls (*n* = 13) matched for age and sex, without personal or family history of epilepsy (up to second degree relatives) or other neurologic/psychiatric disorder(s).

### Histological Processing and Immunohistochemical Reactions

Histological sections (4 μm thick) were submitted to routine staining (hematoxylin and eosin) or immunohistochemical reactions. For immunostaining, the sections were incubated with primary antibodies for 18 h at 4°C and, then, the detection system containing secondary antibody and peroxidase (AdvanceTMHRP®, Dako, cat # K4068, Glostrup, Denmark) was added for 30 min at 37°C. 3,3′-Diaminobenzidine (DAB) was used as chromogenic substrate. Specifically, NeuN antibody (1:1000, Merck Millipore®, cat # MAB377, A60, Billerica, MA, USA) was employed to evaluate heterotopic neurons. Ki67 marker (1:500, Dako/Agilent®, cat # M7240, MIB-1, Santa Clara, CA, USA) was used to assess cellular proliferative activity. CD34 antibody (1:100, Dako/Agilent®, cat # GA632, QBEnd 10, Santa Clara, CA, USA) was used to detect immature cells. Olig2 staining (1:500, Merck Millipore®, cat # MABN50, 211F1.1, Billerica, MA, USA) was performed to identify oligodendroglial cells. CD45 antibody (1:100, Dako ®, cat # GA751, 2B11+PD7/26, Glostrup, Denmark) was used to identify lymphoid cells. Myeloarchitecture integrity was accessed by means of 2′, 3′-cyclic-nucleotide 3'-phosphodiesterase (CNPase) immunostaining (1:500, cat # MAB326, 11-5 B, Millipore®, Darmstadt, Germany) (15). C-fos (1:100, Abcam® cat# ab208942, 2H2, Cambridge-MA, USA) and ΔFosB (1:100, Abcam®, cat # EPR15905, ab184938, Cambridge-MA, USA) antibodies were used as markers of acute and chronic neuronal activity, respectively (16–19).

For each marker, immunohistochemical reactions were concomitantly performed in all samples to ensure similar technical conditions. External control sections were simultaneously run to confirm the success of the immunostaining.

### Histopathological Analysis

All cell counts in either hematoxylin and eosin-stained or immunostained sections were performed at two levels of the WM, as previously defined (15). Particularly, the superficial white matter (SWM) was defined as <500 μm from the cortical junction, and the deep white matter (DWM) as more than 500 μm distant from the cortical junction. For each level (SWM and DWM), 5 randomly chosen high-power fields (HPF; 400x; objective field area equivalent to 0.092 mm^2^) were photographed (Capture system: HD Lite Tucson 1080p; Microscope Novel BM2100). Subsequently, the number of cell types (or immunopositive) was obtained in each photodocumented field using the “cell counter” function of the ImageJ® software (version 1.50i, National Institutes of Health-US, https://www.nih.gov). The random choice of the histological fields and the cell counts were performed in agreement by two neuropathologists (BCZ and FR).

### Analysis of CNPase Immunostaining

CNPase immunostaining was assessed in slides without counterstaining and employing the ImageJ® software to increase binary contrast (thresholding tool). HPFs of both SWM and DWM levels were randomly selected as described above (item Histopathological Analysis). Briefly, photomicrographs of each HPF were initially transformed into gray scale and then converted into binary information. For such conversion, the software operator visually defined a cutoff point in the gray scale that maintained, as reliably as possible, the immunostaining features observed in the color images (intensity and contrast with the background). Particularly, this gray scale corresponds to numerical values ranging from 0 (black) to 255 (white). Moreover, grayscale values below and above the cut-off point become black and white, respectively. Finally, the software sums the gray values of all pixels in each field and divides by the total number of pixels in the same field, thus, providing a value corresponding to the percentage of immunostained area (% area) (20).

### MRI Acquisition and Processing

We performed a routine MRI protocol for epilepsy cases in a 3T Philips Achieva scanner, as previously described (21, 22). Such protocol included T1-weighted 3D images, T1 and T2-weighted images in the axial and coronal planes, fluid-attenuated inversion recovery (FLAIR) images, susceptibility weighted imaging (SWI), inversion recovery (IR) coronal imaging, and diffusion-weighted imaging (DWI). We also performed T2-weighted coronal multiecho images for T2 relaxometry analysis (five echo times: 30/60/90/120/150 ms; 3 mm thick, repetition time = 3300 ms; matrix 200 × 176; FOV 1802 × 180 mm^2^). For diffusion parameter analysis, we acquired a spin-echo single-shot echo planar imaging (EPI) (2 x 2 x 2 mm voxel size, interpolated to 1 x 1 x 2 mm; reconstructed matrix 256 x 256; 70 slices; TE/TR 61/8,500 msec; flip angle 90°; 32 gradient directions; no averages; max b-factor = 1,000 s/mm^2^; 6-min scan) and 3D T1-weighted images (1 × 1 × 1 mm, no gap, flip angle = 8°, repetition time [TR] = 7.0 ms, echo time [TE] = 3.2 ms, matrix 240 × 240, field of view (FOV) = 240 mm × 240 mm).

### Relaxometry

**Figure 1:**
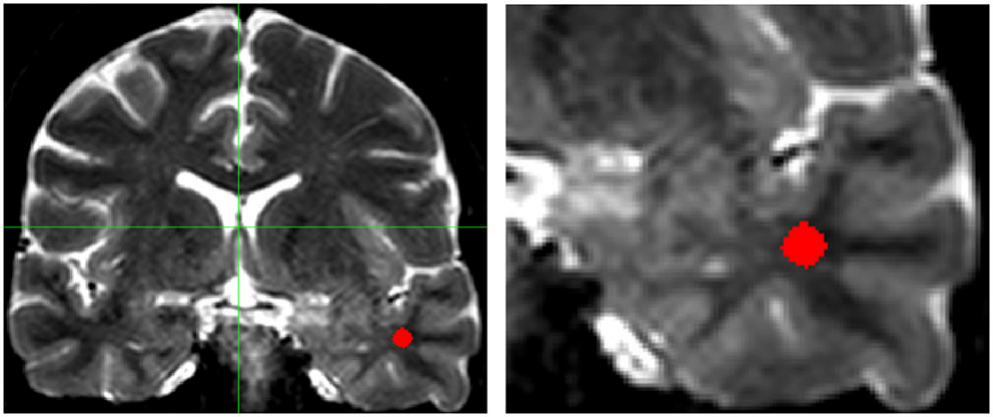
T2-weighted coronal multiecho images showing the localization of the region of interest (ROI) in the white matter of the temporal pole (**Left**: complete scan, **Right**: detail of the scan shown on the left). Areas of artifactual increase in T2-weighted hyperintense signal were carefully avoided. Images are in radiological convention.

We processed the T2-weighted multiecho coronal images of all individuals with the Aftervoxel software (http://www.liv.ic.unicamp.br/b~ergo/aftervoxel) as previously documented (21). The region of interest (ROI) was defined using the postoperative T1-weighted image as a visual control to localize the surgical resected area. Then, we manually drew the ROIs in the corresponding topography on the preoperative T2-weighted multiecho, always in the same echo, selecting three portions of the WM in three consecutive slices from approximately the same area of the temporal pole. To define the ROIs in control subjects, we chose an analogous topography to that of the matched patient (anterior temporal pole, ipsilateral to the postoperative area). The ROIs were drawn avoiding areas of artifactual T2 hyperintensity and interference in signal values caused by incorrect demarcation of cerebrospinal fluid in the structures. We carefully drew WM areas of equivalent size across all individuals (Figure 1).

### Diffusion Parameters Analysis

**Figure 2:**
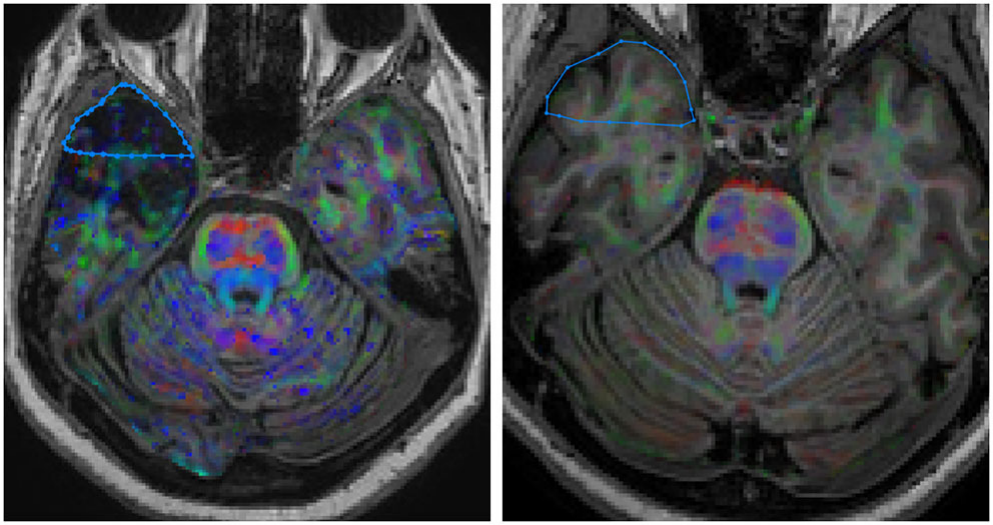
DWI coregistered to the volumetric T1-weighted images showing the localization of the region of interest (ROI) in the white matter of the temporal pole. **(Right)** HS group; **(Left)** control group. Images are in neurological convention.

We used DWI and T1-weighted imaging to examine differences in macro and microstructural integrity of the WM. The diffusion maps in the ROIs were obtained using the ExploreDTI software (A. Leemans, University Medical Center, Utrecht, The Netherlands) (23). First, we registered the preoperative 3D T1-weighted with the postoperative 3D T1-weighted images, using the anterior commissure as an anatomical landmark, as described elsewhere (22). The preoperative DWI was then registered with the resulting coregistered T1-weighted 3D images to ensure the best overlap and define the WM area surgically resected. Then, the axial section where the surgical site presenting the largest dimensions was identified, and the WM ROI was manually drawn. The DWIs were corrected for eddy currents and Gibbs ringing. The susceptibility artifacts were attenuated during non-linear registration using volunteers T1 WI, according to ExploreDTI recommended procedures. We performed the same protocol for controls, defining the ROI in a similar topography, ipsilateral to the surgery of the matched patients. From each ROI, we obtained the mean values of the parameters fractional anisotropy (FA), mean diffusion (MD), axial diffusion (AD), and radial diffusion (RD) (24) (Figure 2).

### MRI Analysis

Magnetic resonance imaging were retrospectively reanalyzed by two experienced neuroimagers (VCMC and FC) considering both clinical and EEG findings. Nonvolumetric sequences were analyzed first with T1 inversion recovery, followed by T1, T2, FLAIR, and SWI sequences. The evaluation corresponded to visual analysis of 12 parameters based on previously described criteria (25, 26). We paid particular attention to the presence of blurring and atrophy of the ipsilateral temporal pole, which are reported to occur in 28–66% of patients with TLE-HS (12, 27, 28).

### Statistical Analysis

Statistical analyses were performed using SAS 9.2 Software for Windows (SAS Institute Inc, 2002–2008, Cary, NC, USA). The data were analyzed using Fisher exact test for categorical variables and Mann–Whitney test to evaluate differences in MRI variables between groups. Then, we applied receiver operating characteristic (ROC) curves to test whether significant MRI and histopathological metrics were sensitive and specific to alterations in the WM of individuals with TLE-HS. Also, we obtained the best cut-off values optimizing sensitivity and specificity, therefore, indicative of pathological changes. To study the relationship between histopathological and MRI data in the TLE-HS group, Spearman's correlation coefficients were calculated. Finally, we plotted ROC curves to assess whether cell count, immunostaining, and neuroimaging data discriminate TLE–HS group from controls, obtaining a cut-off value associated with a higher probability of changes observed in HS patients. To minimize multiple comparisons, we included in the correlation and ROC analyses the histopathological findings and MRI features considered to be relevant to the group analysis (please refer to the Results section). Data were reported as mean ± standard deviation (SD), median and interquartile interval, and percentages, as appropriate. The significance level adopted for the statistical tests was 5%.

## Results

### Clinical Data

Nine individuals with ILAE type 1 HS were studied, their ages at surgery ranging from 39 to 56 years old (mean age ± standard deviation: 47.00 ± 6.67 years). Six patients were women (66.66%) and three were men (33.33%). The average preoperative interval during which seizures occurred was 36.64 years (±11.02), with a minimum of 21 years and a maximum of 50 years. The mean age at seizure onset was 10.4 years (±11.05), ranging from 2 months to 35 years. Seven patients showed fluorodeoxyglucose positron emission tomography (FDG-PET) hypometabolism in the temporal pole (data not available for two patients). Regarding postoperative seizure control [Engel's class (29)], 5 patients (55.55%) were classified as IA (completely seizure-free since surgery), 1 (11.11%) was classified as IIA (initially free of disabling seizures but has currently rare seizures), and 3 (33.33%) were grouped as IIIA (worthwhile seizure reduction). The average postoperative follow-up was 2.17 years. Further details on clinical and EEG findings are presented in Table 1.

**Table 1:** Summary of clinical data and descriptive MRI parameters in patients with HS.

**Patient #**	**Gender**	**Side of resection**	**Age at onset (years)**	**Age at surgery (years)**	**Epilepsy duration**	**Engel's class**	**Interictal EEG**	**Ictal EEG**	**Temporal lobe asymmetry**	**T2/FLAIR Temporopolar blurring**
1	F	Left	13	51	38	Ia	Left temporal	Left temporal	Present	Absent
2	F	Right	0.17	38	37.89	IIIa	Bilateral temporal (R > L)	Bilateral fronto-temporal (R > L)	Present	Present
3	M	Left	2	51	49	IIa	Left temporal	Bilateral temporal (L > R)	Absent	Absent
4	F	Left	5	44	39	Ia	Left temporal	Left temporal	Present	Present
5	M	Left	3	49	46	IIIa	Bilateral temporal (L > R)	Bilateral temporal (L > R)	Present	Present
6	F	Right	18	41	23	Ia	Right temporal	Right temporal	Absent	Absent
7	F	Right	35	56	21	IIIa	Bilateral temporal (R > L)	Right temporal	Absent	Absent
8	F	Left	4	54	50	Ia	Bilateral temporal (L > R)	Left temporal	Absent	Present
9	M	Left	13	39	26	Ia	Left temporal	Left temporal	Absent	Present

As regards the control group for histopathological analyses (*n* = 10; five women and five men), the mean age at autopsy was 43.00 ± 12.38 years (ranging from 23 to 67 years). For MRI control group (*n* = 13; eight women and five men), the mean age at acquisition was 52.54 ± 8.96 years (ranging from 36 to 65 years). There was no statistical difference regarding age when the HS group was compared to autopsy and MRI controls (*p* = 0.326 and *p* = 0.101, respectively).

### Quantitative Analysis of Cell Population in the White Matter

**Figure 3:**
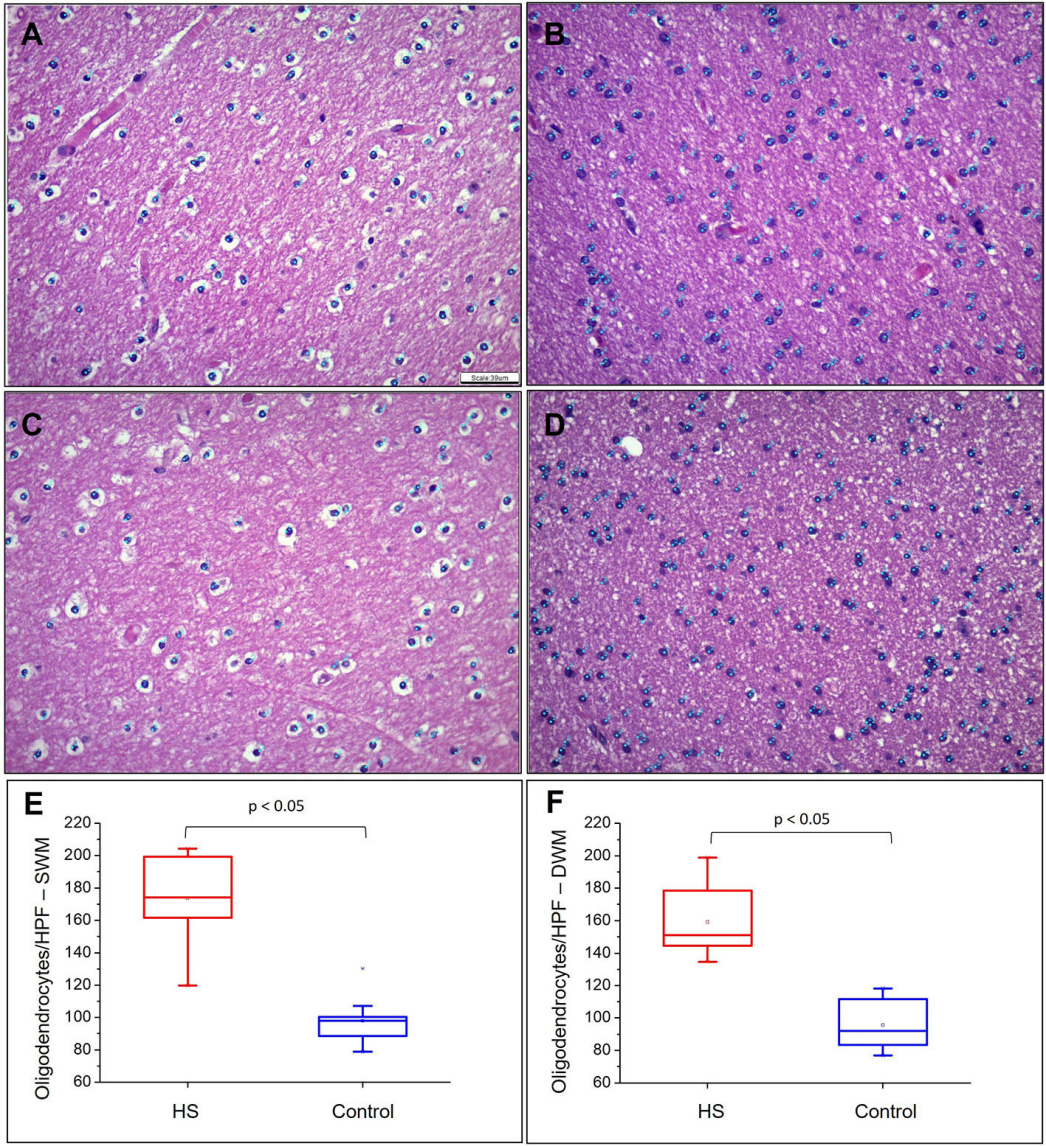
Quantitative analysis of the number of oligodendrocytes in the superficial white matter (SWM) **(A)** control and **(B)** HS group; and in the deep white matter (DWM) **(C)** control and **(D)** epilepsy group. Graphical representation of the mean number of oligodendroglial cells in SWM **(E)** and DWM **(F)**. Note the identification of cells counted in each field by means of blue dots corresponding to the “cell counter” counting tool of the ImageJ® software. Hematoxylin and eosin **(A–D)**. Scale bar: 39 μm **(A–D)**.

**Figure 4:**
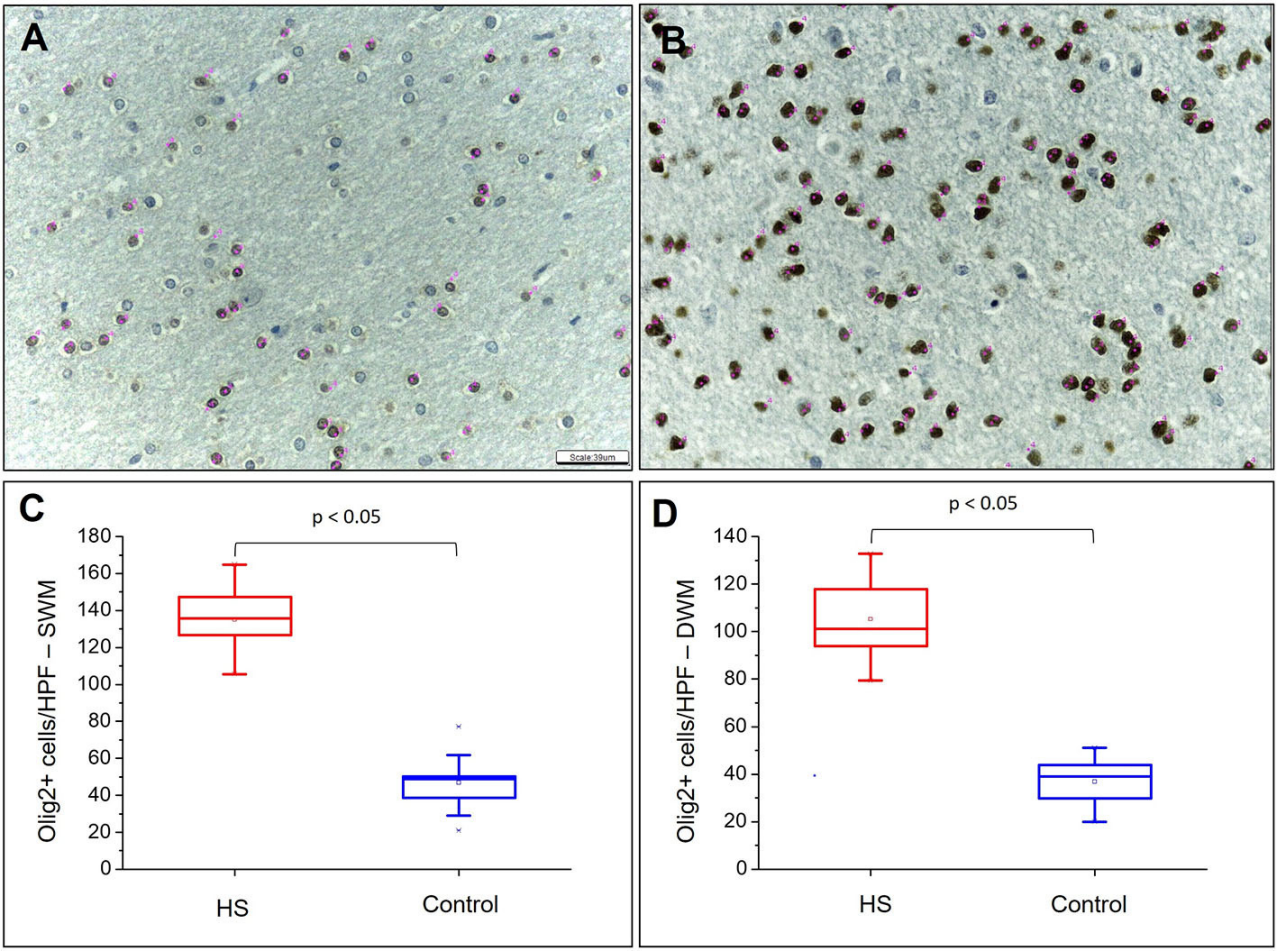
Quantitative analysis of the immunohistochemical expression of Olig2. Olig2 expression in control **(A)** and HS **(B)** individuals. Graphic representation of the average number of cells with Olig2 expression in SWM **(C)** and DWM **(D)**. Note the identification of cells counted in each field by means of pink dots corresponding to the “cell counter” counting tool of the ImageJ® software. Peroxidase **(A, B)**. Scale bar: 39 μm **(A, B)**.

**Figure 5:**
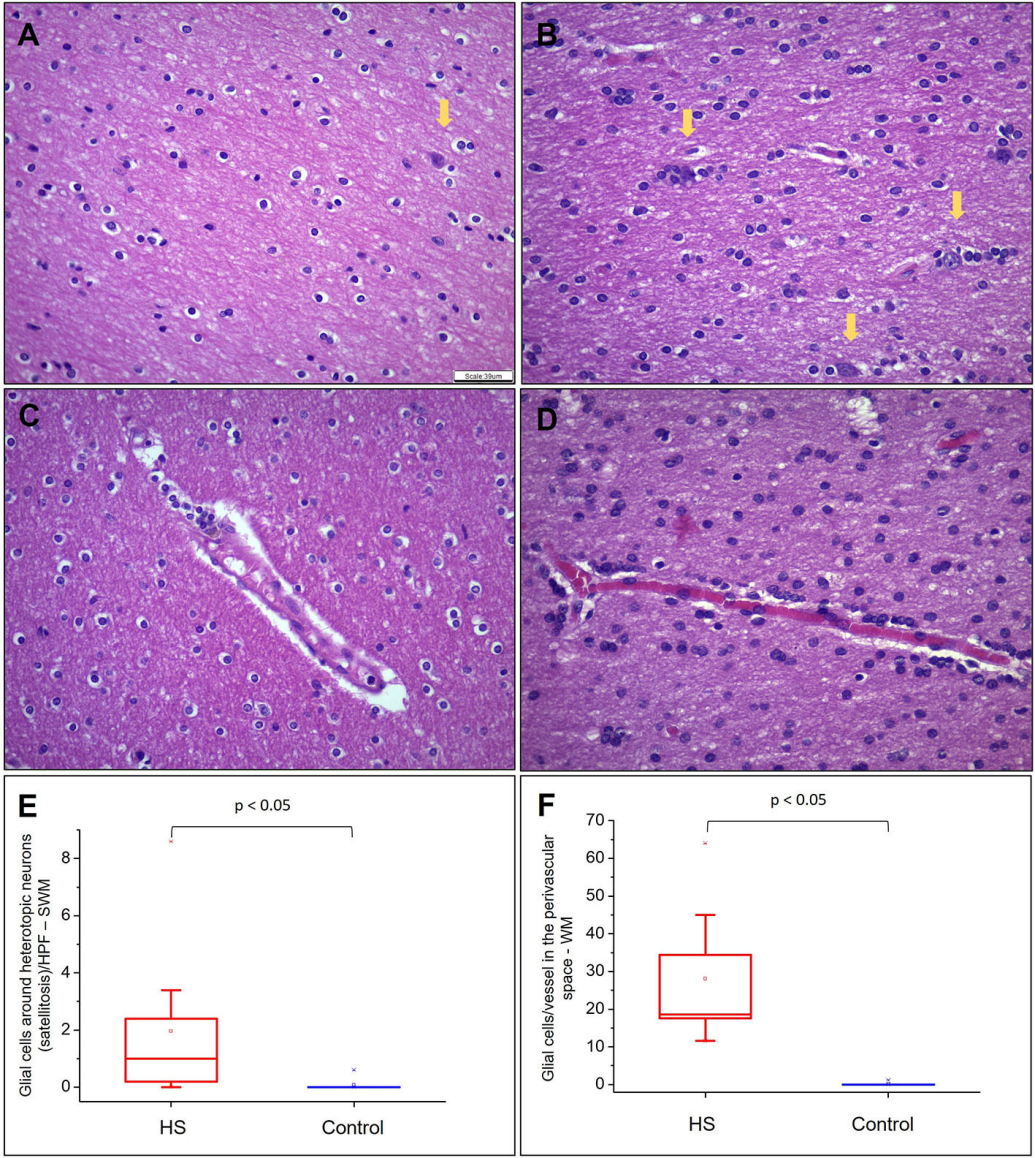
Quantitative analysis of heterotopic neurons, satellitosis, and glial cells in perivascular space in WM. **(A)** and **(B)** represent fields photographed in the SWM of a control subject and an HS patient, respectively. Heterotopic neurons are indicated with yellow arrows. **(C)** and **(D)** represent glial cells in the perivascular space in a control group and an HS group, respectively. In **(E)**, graphical representation of the mean number of oligodendroglial cells around heterotopic neurons by HPF in SWM. In **(F)**, mean number of glial cells in the perivascular space/vessel in each studied group. Hematoxylin and eosin **(A–D)**. Scale bar: 39 μm **(A–D)**.

Hematoxylin and eosin-stained slides from the anterior pole of the temporal lobe from individuals with HS showed a significant increase in the number of oligodendroglial cells per HPF, both in the SWM and DWM, in comparison with controls (HS – SWM: 173.53 (mean) ± 27.87 (standard deviation) and DWM: 159.20 ± 21.56; Control (C) – SWM: 97.88 ± 14.01 and DWM: 95.56 ± 16.06; *p* < 0.001) (Figure 3). Such increase was also detected using the Olig2 antibody (HS – SWM: 135.02 ± 19.97, DWM: 105.31 ± 16.52; C – SWM: 46.82 ± 15.78, DWM: 36.80 ± 10.42; *p* < 0.001) (Figure 4). In addition, the HS group presented a significant increase in the number of perivascular oligodendroglial cells per vessel (HS – 28.09 ± 17.14; C – 0.16 ± 0.39; *p* < 0.001) or adjacent to heterotopic neurons (perineuronal satellitosis; HS – SWM: 1.96 ± 2.75, DWM: 0.64 ± 0.55; C – SWM: 0.08 ± 0.19; *p* = 0.008, DWM: 0.04 ± 0.13; *p* = 0.001) (Figure 5).

**Figure 6:**
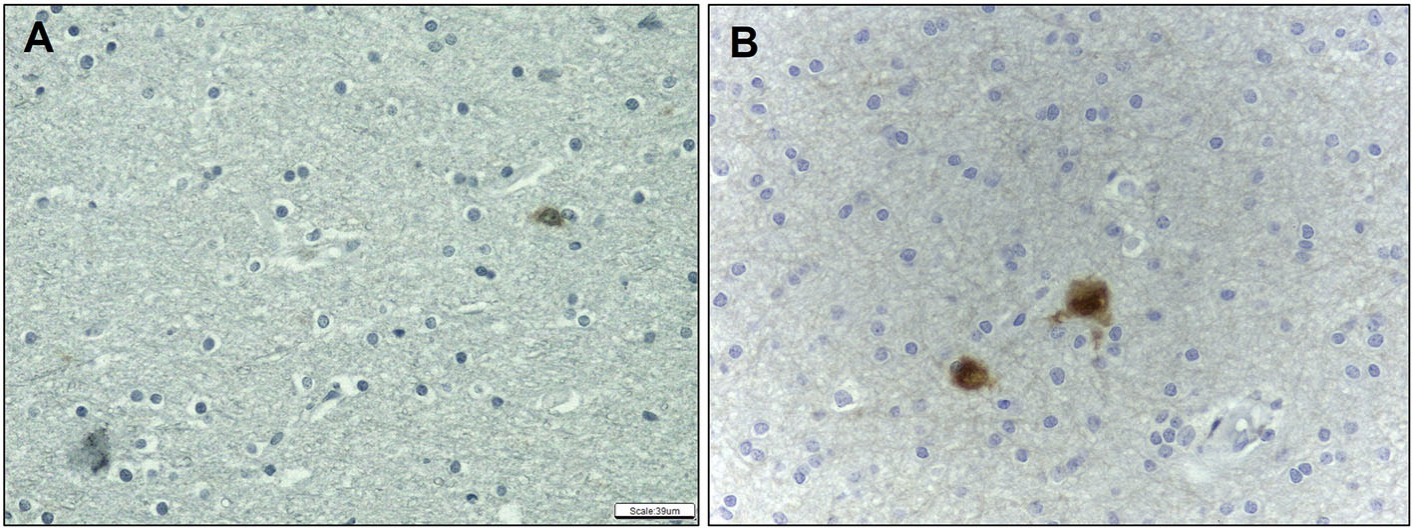
Quantitative analysis of the immunohistochemical expression of NeuN antibody. NeuN expression in control **(A)** and HS **(B)** individuals. Peroxidase **(A, B)**. Scale bar: 39μm **(A, B)**.

As regards heterotopic neurons, we observed an increase in their frequency in the SWM of HS samples only after labeling with NeuN in comparison with controls (HS – SWM: 1.47 ± 0.46; C – SWM: 0.70 ± 0.29; *p* = 0.002) (Figure 6). Particularly, no significant difference was noted in such cell count on hematoxylin and eosin-stained sections both in SWM (HS – 0.71 ± 0.66; C – 0.28 ± 0.21; *p* = 0.171) and DWM (HS – 0.33 ± 0.36; C – 0.10 ± 0.22; *p* = 0.051). Likewise, there was no difference between the groups regarding the number of NeuN-positive neurons in the DWM (HS – 0.47 ± 0.22; C – 0.32 ± 0.30; *p* = 0.151).

Neither chronic inflammation nor CD45-positive cells were identified in the groups. Immunostaining for CD34 was verified only in the vascular endothelium (no immature CD34-positive cells were observed), irrespective of epilepsy history.

### Tissue Expression of CNPase and Ki67 Labeling

**Figure 7:**
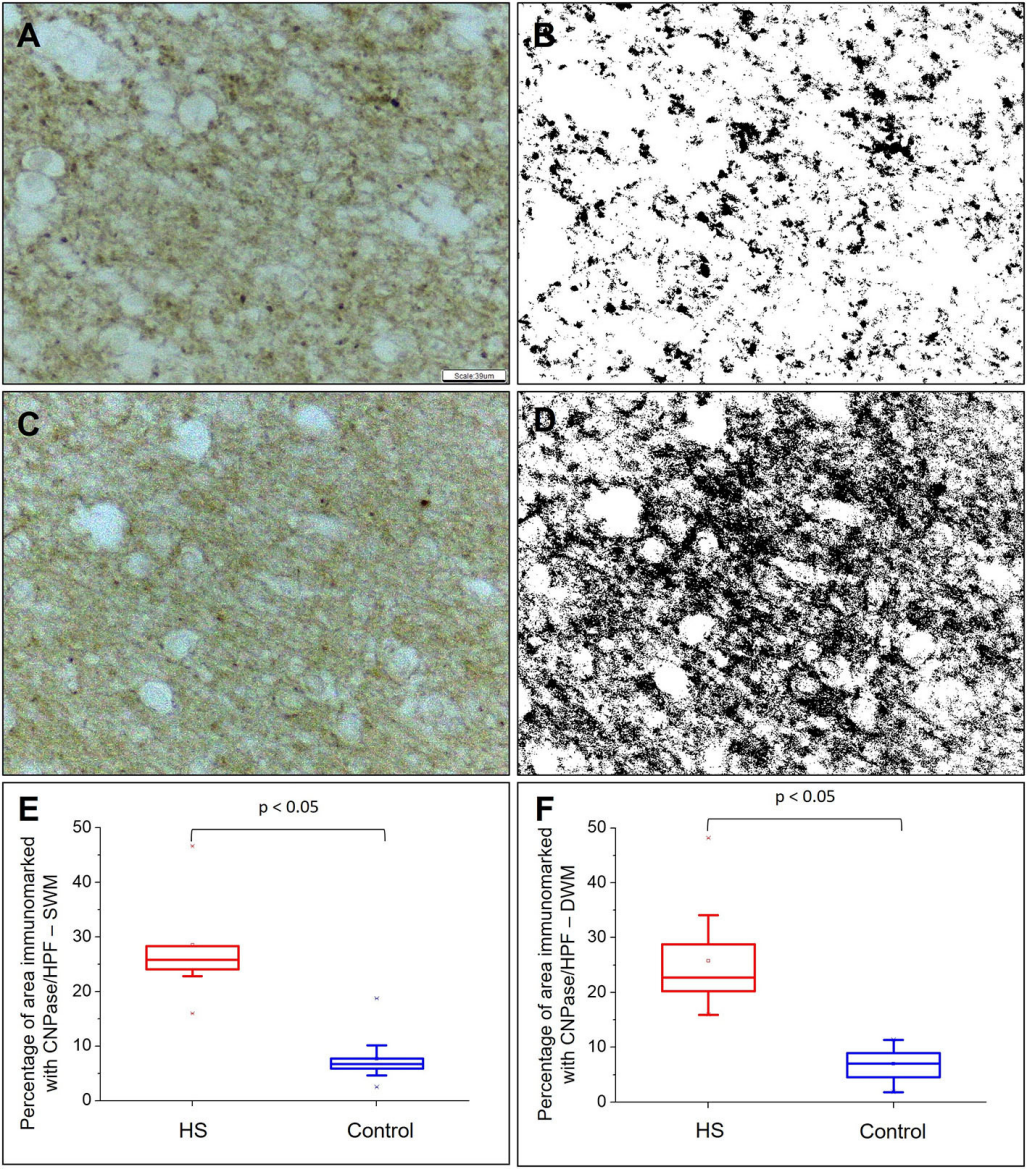
Quantitative analysis of myeloarchitecture through CNPase expression. CNPase expression without counterstaining in control **(A)** and epilepsy **(C)** groups. In **(B)** and **(D)**, transformation of images **(A)** and **(C)** are shown, respectively, in gray scale to increase binary contrast (thresholding) and analysis of the percentage of immunomarked area using the ImageJ® program. Graphic representation of the mean percentage of CNPase expression per high power field (HPF) in superficial white matter **(E)** and deep white matter **(F)**. Peroxidase **(A, C)**. Scale bar: 39 μm **(A–D)**.

The percentage of CNPase-immunopositive area was significantly higher both in the SWM and DWM in patients with HS compared with controls (HS – SWM: 28.51 ± 9.58%, DWM: 25.80 ± 10.06%; C – SWM: 7.66 ± 4.35%, DWM: 7.02 ± 3.09%; *p* < 0.001) (Figure 7).

Proliferative activity, as shown by nuclear staining for Ki67, was significantly higher in the DWM of the HS group (HS – 0.44 ± 0.42, C – 0.04 ± 0.13; *p* = 0.005), but not in SWM (HS – 0.18 ± 0.27, C – 0.02 ± 0.06; *p* = 0.091).

### Tissue Expression Analysis of Acute and Chronic Neuronal Markers

**Figure 8:**
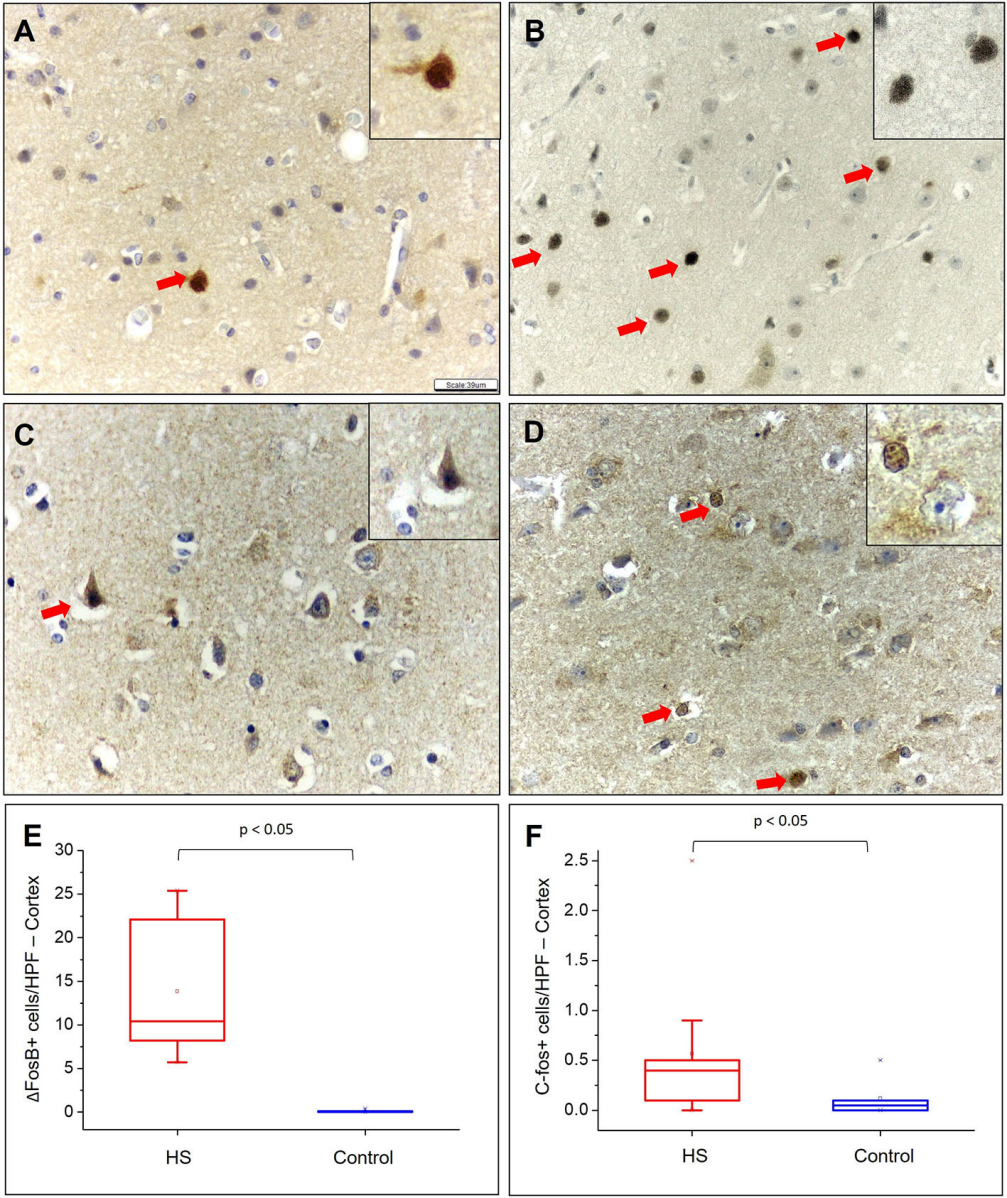
Quantitative analysis of the immunohistochemical expression of ΔFosB and c-fos. Expression of ΔFosB in the cortical region of individuals in the control **(A)** and HS **(B)** groups. C-fos expression in the cortical region of individuals in the control group **(C)** and in the HS group **(D)**. Neurons with expression of ΔFosB and c-fos are indicated with red arrows. Graphic representation of the average number of ΔFosB **(E)** and c-fos **(F)** positive cells/HPF in neocortex. Peroxidase with hematoxylin counterstaining **(A–D)**. Scale bar: 39μm **(A–D)**.

We also studied markers of acute (c-fos) and chronic (ΔFosB) neuronal activity in both the groups. Specifically, there was a significant increase in neocortical immunolabeling for c-fos (HS – 0.57 ± 0.77; C – 0.12 ± 0.18; *p* = 0.004) and ΔFosB (HS – 13.86 ± 7.54; C – 0.07 ± 0.13; *p* < 0.001) in patients with HS (Figure 8).

### MRI Findings

The parameters assessed in MRI scans and their corresponding frequencies in the HS group are depicted in Table 2. Specifically, there was a significant increase in T2 signal values (relaxometry) in patients with epilepsy when compared with controls (*p* = 0.002). Regarding DWI, we found significantly reduced FA values in the HS group (*p* = 0.034), but no significant differences for AD (*p* = 0.185), RD (*p* = 0.064), or MD (*p* = 0.093).

**Table 2:** Summary of the MRI measurements of the anterior temporal lobe in control group and HS group.

**MRI measurements**	**Control**	**HS**	***p*-value**
T2 relaxometry, median (interquartile range)	68.75 (68.71–70.57)	77.51 (75.53–86.31)	0.002
FA, median (interquartile range)	0.39 (0.27–0.4)	0.24 (0.22–0.243)	0.034
AD, median (interquartile range)	0.00095 (0.00091–0.00122)	0.00121 (0.00113–0.00123)	0.185
RD, median (interquartile range)	0.0007 (0.0005–0.0008)	0.00088 (0.00079–0.0009)	0.064
MD, median (interquartile range)	0.00078 (0.00066–0.00098)	0.00099 (0.0009–0.00102)	0.093

**Figure 9:**
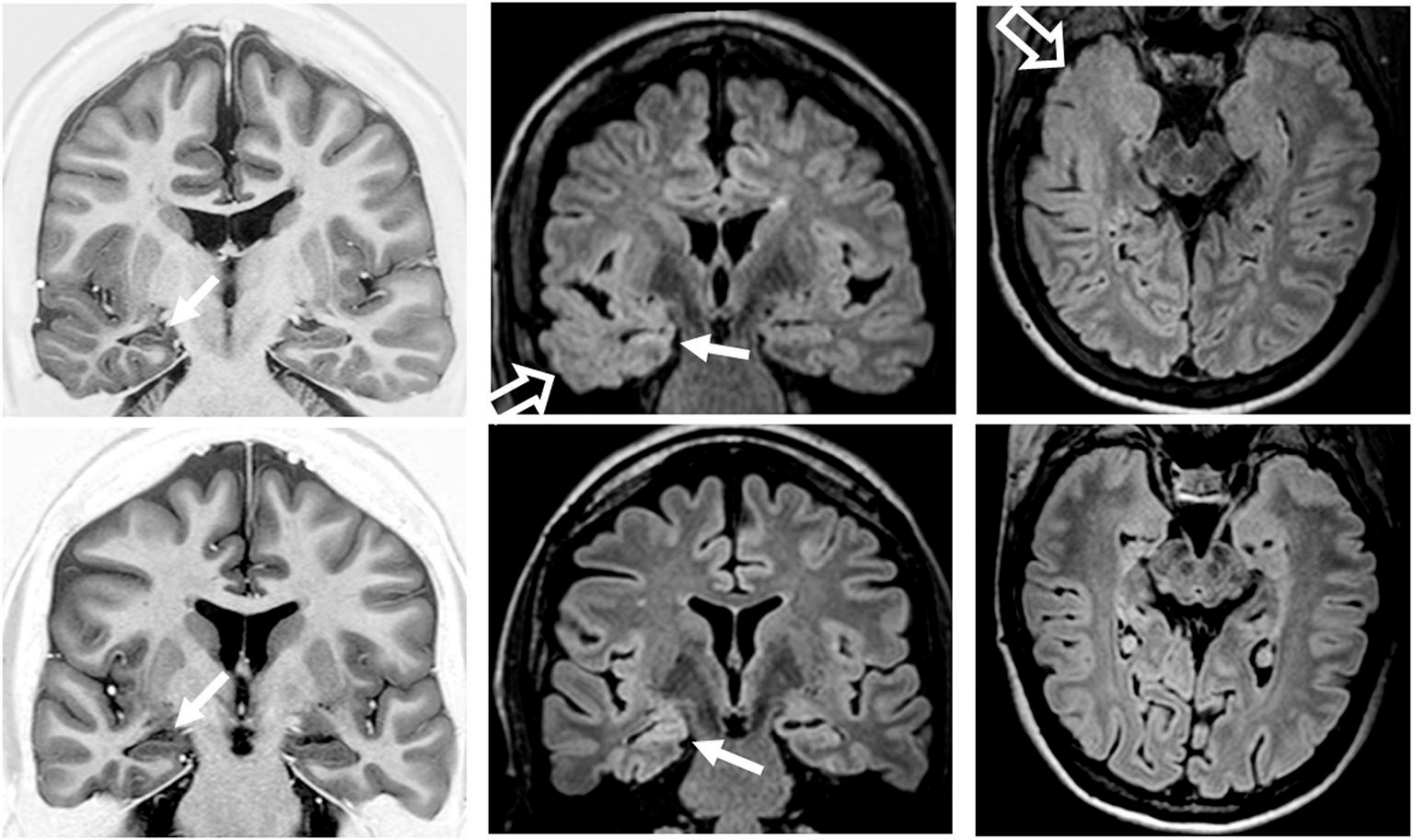
Preoperatory coronal T1-weighted inversion recovery and coronal and axial fluid-attenuated inversion recovery (FLAIR) images from patient 2 **(top row)** with temporopolar blurring in FLAIR images (open arrows) and from patient 6 **(bottom row)** without temporopolar blurring. Both patients had MRI signs of right hippocampal sclerosis (atrophy and hyperintense FLAIR signal; arrows).

All patients had classical MRI signs of unilateral HS in the visual analysis (Figure 9). Temporopolar blurring in T2-weighted/FLAIR images was present in five out of nine patients. Temporal lobe asymmetry was observed in four out of nine patients. One patient also had a small encephalocele.

### Analyses of Association Between Clinical Data, Cell Count, MRI Findings, and Immunohistochemical Expression

**Figure 10:**
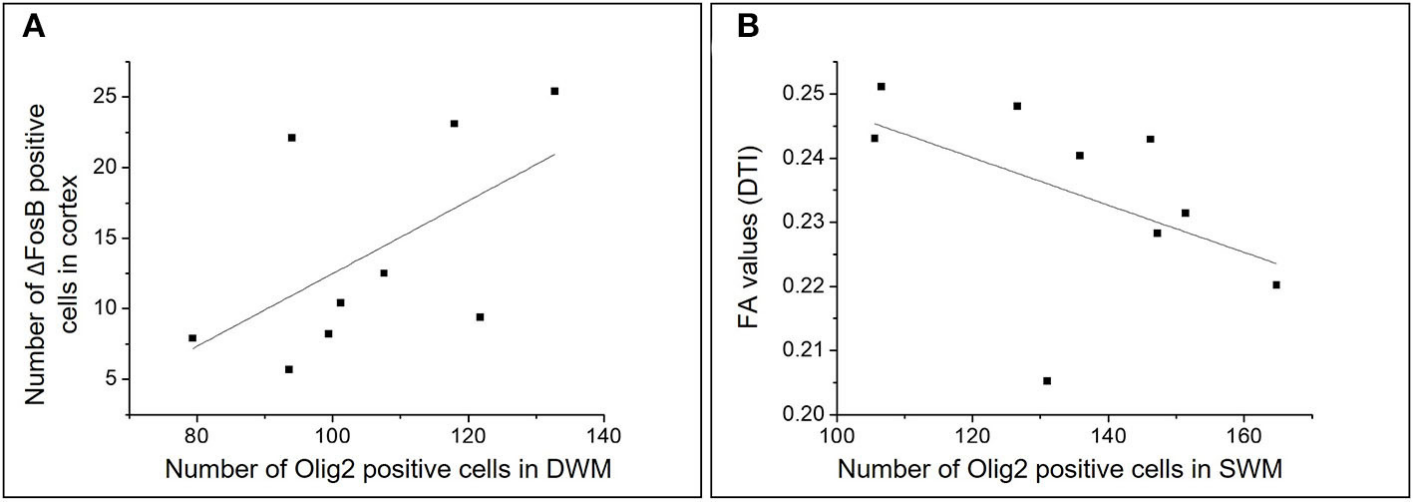
Graphic representations of the analysis of correlations between different numerical variables in the HS group. Scatterplots representing the correlations between the number of ΔFosB positive cells in the cortex and the number of Olig2 positive cells in the DWM **(A)** and between the number of Olig2 positive cells in the SWM and the mean FA in individuals with HS **(B)**.

A positive correlation was found between the number of cells expressing the chronic neuronal activity marker (ΔFosB) in the cortex and the number of Olig2-positive cells in the DWM (*r* = 0.700; *p* = 0.036) (Figure 10).

Moreover, there was a negative correlation between the number of Olig2-positive cells and the FA in the SWM (*r* = −0.666; *p* = 0.049) and a non-significant correlation in DWM (*r* = −0.250, *p* = 0.51) (Figure 10). We also found a negative correlation between the number of cells expressing ΔFosB in the SWM and the FA mean in patients with HS (*r* = −0.788; *p* = 0.012).

There were no significant correlations between oligodendrocyte count (hematoxylin and eosin-stained sections) and T2-weighted signal measured by relaxometry in both the SWM (−0.083, *p* = 0.83) and DWM (*r* = −0.500, *p* = 0.17); oligodendrocyte count and FA in the SWM (*r* = −0.0833, *p* = 0.83) and DWM (*r* = −0.183, *p* = 0.63); CNPase-positive area (%) and T2 signal in the SWM (*r* = 0.017, *p* = 0.96) and DWM (*r* = −0.283, *p* = 0.46); and CNPase-positive area and FA in the SWM (*r* = 0.317, *p* = 0.41) and DWM (*r* = 0.001, *p* = 1.00).

When comparing patients with and without temporal polar blurring in the visual analysis of T2-weigthed and FLAIR images, we did not find significant differences (*p* > 0.1) in the number of oligodendroglial cells (hematoxylin and eosin-stained sections and Olig2-positive) and CNPase-immunopositive areas. Moreover, there were no significant correlations (*p* > 0.1) when considering the number of oligodendroglial cells or CNPase-immunopositive areas and duration of epilepsy.

### Discrimination of Study Groups Using ROC Curves

The ROC analyses showed that the number of oligodendroglial cells (hematoxylin and eosin-stained sections) differentiated patients with HS both in the SWM (AUC = 0.989, 95%CI 0.954–1.024; *p* < 0.001) and DWM (AUC = 1, 95%CI 1–1; *p* < 0.001). Particularly, the cut-off point for cells in the SWM was ≥113 cells/HPF with sensibility = 100.0% (95%CI 62.88–100.0) and specificity = 90% (95%CI 54.11–99.48). Regarding the DWM, the cut-off point was ≥126 cells/HPF with sensibility = 100.0% (95%CI 62.88–100.0) and specificity = 100% (95%CI 62.55–100.0). Similarly, the number of oligodendroglial cells in the perivascular space (AUC = 1, 95%CI 1–1; *p* < 0.001) differentiated the HS group. In this case, the cut-off point was ≥6 cells/vessel with sensibility =100.0% (95%CI 62.88–100.0) and specificity = 100.0% (95%CI 65.55–100.0).

As regards immunostaining, we found that the number of NeuN-positive heterotopic neurons in the SWM differentiated the HS group (AUC = 0.917, 95%CI 0.783–1.050; *p* < 0.002) with a cut-off point ≥1.3 NeuN-positive cells/HPF with sensibility = 77.78% (95%CI 40.19–96.05) and specificity = 100.0% (95%CI 65.55–100.0). Also, Olig2-positive cells differentiated patients with HS both in the SWM (AUC = 1, 95%CI 1–1; *p* < 0.001) and in the DWM (AUC = 1, 95%CI 1–1; *p* < 0.001), cut-off points being ≥91 cells/HPF with sensibility = 100.0% (95%CI 62.88–100.0) and specificity = 100.0% (95%CI 65.55–100.0); and ≥65 cells/) and specificity = 100.0% (95%CI 79.95–100.0), respectively.

The percentage of area immunoreacted to CNPase also differentiated the HS group, both in the SWM (AUC = 0.989, 95%CI 0.954–1.024; *p* < 0.001) and in the DWM (AUC = 1, 95%CI 1–1; *p* < 0.001). The cut-off points were ≥13%/HPF in the SWM with sensibility = 100.0% (95%CI 62.88–100.0) and specificity = 90.0% (95%CI 54.11–99.48) and ≥13.6%/HPF in the DWM with sensibility = 100.0% (95%CI 62.88–100.0) and specificity = 100.0% (95%CI 65.55–100.0), respectively.

The neocortical markers of acute and chronic neuronal activity could also discriminate the patients with HS (c-fos–AUC = 0.778, 95%CI 0.563–0.992, *p* = 0.041; ΔFosB – AUC = 1, 95%CI 1–1, *p* < 0.001). For c-fos, the cut-off point was ≥0.15 cells/HPF with sensibility = 66.67% (95%CI 30.92–90.96) and specificity = 80.0% (95%CI 44.22–96.46). As for ΔFosB, the cut-off point was ≥3.0 cells/HPF with sensibility = 100.0% (95%CI 62.88–100.0) and specificity = 100.0% (95%CI 65.55–100.0).

The T2 signal by relaxometry significantly differentiated individuals with HS from controls (AUC = 0.926, 95%CI 0.809–1.043, *p* = 0.002), cut-off being ≥70.6 with sensibility = 88.89% (95%CI 50.67–99.42) and specificity = 77.78% (95%CI 40.19–96.05).

The FA could also significantly discriminate the HS and control groups (AUC = 0.802, 95%CI 0.555–1.050; *p* = 0.031). The cut-off was ≤ 0.26 with sensibility =100% (95% CI 62.88–100.0) and specificity = 77.78% (95%CI 40.19–96.05).

## Discussion

This work aimed to investigate and correlate WM alterations in cell populations and neuroimaging from individuals with a common cause of pharmacoresistant epilepsy (HS), and whose surgical treatment allowed the sampling of tissue from the temporopolar region. Specifically, we demonstrated significant changes in WM cellularity when analyzing the anterior temporal pole of patients with HS. Additionally, histopathological alterations of the anterior temporal pole, in particular related to the oligodendrocyte population were correlated with MRI findings, allowing to determine cut-off points that could predict changes associated with HS and hypothesize pathophysiological mechanisms for TLE–HS.

Oligodendroglial hypercellularity in the WM was an eye-catching finding in the present investigation. Such a finding has been observed in patients with long-term epilepsy and reported under a wide range of descriptive terms, such as glioneuronal hamartia (30), microdysgenesis (8, 9, 31), oligodendroglial hyperplasia (10, 15), and oligodendrogliosis (32, 33). Recently, an entity was proposed in patients with early-onset frontal lobe epilepsy and designated as mild developmental cortical malformation with oligodendroglial hyperplasia (34, 35). This myriad of terms has been used for patients with temporal (8, 9, 30, 36, 37) or frontal lobe epilepsy (15, 34) and associated with different pathological conditions (32).

Changes related to the number and distribution of oligodendrocytes in the WM have been interpreted as a reactive proliferation in the context of pharmacoresistant epilepsy (32, 36). Experimental studies on optogenetic stimulation in mice showed evidence supporting this interpretation. Indeed, it was demonstrated that repetitive activation of neurons is capable of stimulating the proliferation of both neuronal and oligodendroglial progenitor cells, their differentiation, and myelination process (11). We verified an increased expression of acute and chronic markers of neuronal activity in the temporal neocortex. This finding suggests that the adaptive neuroplasticity described in experimental models of epilepsy can also be observed in human samples from patients with TLE-HS (11, 18, 19). Particularly, as observed in experimental studies (19), the increased expression of ΔFosB that we verified in patients with TLE could be associated with higher expression of specific subunits of glutamate receptors, thus contributing to seizure generation and propensity to seizures. Moreover, we also verified that increased neuronal activation was positively correlated with oligodendroglial hypercellularity in the WM, even though such increase in cell number was not correlated with disease duration. However, since our patients had more than 21 years of epilepsy duration (ranging from 21 to 50 years), a floor effect should be considered in this particular lack of correlation. Therefore, considering the observations from previous reports on surgical specimens and experimental approaches and the current findings in the temporal pole, it is possible that the alterations related to oligodendroglial cell number and distribution in the WM may be a maladaptive response of the nervous tissue submitted to pharmacoresistant epilepsy, irrespective of etiology.

In this work, the HS group showed an increase in the percentage of the area immunostained for CNPase. This marker is a myelin membrane-associated protein expressed by both oligodendroglial progenitor forms and mature oligodendrocytes, its function being still debatable (38, 39). As CNPase is considered to bind to microtubules and to be involved in axonal maintenance, this protein would play a structural role in myelin integrity (40). Therefore, we hypothesize that the increase in the percentage of CNPase immunostained area could be due to fragmentation or structural disorganization of WM fibers in the context of chronic epilepsy (15, 41).

Additionally, we demonstrated a significant ipsilateral reduction in FA in the anterior pole of the temporal lobe, corresponding to a decrease in the integrity of the axonal bundles, and increased T2-weighted signal. This result is in line with previous DTI (42, 43) and T2-weighted reports (21, 22) of widespread changes in TLE–HS affecting not only the hippocampal formation but also other temporal and extratemporal structures. However, fewer studies have investigated the histopathological correlation of WM alterations with imaging in patients with epilepsy.

Overall, FA changes has been more frequently associated with histopathological changes in the SWM. Here, we found that higher numbers of Olig2- and ΔFosB-positive cells were strongly associated with lower FA values, explaining over 44% of the variation between these measures. Concha et al. (42) showed lower FA associated with reduced cumulative axonal circumference, axonal density, and myelin thickness and area in the fimbria–fornix tract, thus evidencing the relationship between diffusion microstructure abnormality and underlying axonal pathology. Interestingly, a recent *in vivo* MRI study (44) also demonstrated that reduced FA was associated with reduced intracellular volume fraction (an MRI marker of neurite density) and myelin water fraction (an MRI marker of myelination). Taken together, it is possible that our findings regarding oligodendrocyte hypercellularity, neocortical neuronal activation, and increase in percentage of CNPase-positive area would suggest that the axonal impairment measured by the FA is part of a complex mechanism involving excessive neuronal activity associated with changes in the myeloarchitecture integrity in patients with TLE.

Moreover, we did not find a significant correlation between T2-weighted signal (relaxometry) and the number of oligodendrocytes in the DWM in hematoxylin and eosin-stained sections. In TLE, a hyperintense signal in T2-weighted images has been associated with astrocytic reaction (gliosis) in the hippocampus (45). Therefore, our findings suggest that tissue changes associated with the oligodendrocyte population in the WM may not be the predominant pathophysiological events contributing to changes in T2 hyperintense signal detected in the temporal pole of some patients with TLE. However, these findings need further confirmation.

Our results regarding visual MRI analysis revealed that five out of nine patients presented with temporopolar blurring in T2-weighted and FLAIR images. However, the increase in CNPase-positive areas and in the number of oligodendroglial cells (both in hematoxylin and eosin-stained and in Olig2-immunoreacted sections) were similar in patients with and without temporopolar blurring. These findings support that this MRI temporopolar blurring in patients with TLE–HS is most likely related to delayed myelination and arrest of white matter development (12, 46) and not predominantly influenced by changes in the number of oligodendrocytes.

The value of hippocampal T2 relaxometry TLE investigation is well-documented using both manual and automatic methods (47, 48). Here, we also showed that WM alterations translated by histopathological, FA, and T2 signal changes in the temporal pole allowed for differentiation of patients with HS from controls, with high sensitivity and specificity, pointing out reference cutoffs to indicate pathological changes. In this sense, previous studies have yielded results that allow imaging techniques to anticipate histopathological findings of epileptogenic lesions (47, 49).

Regarding the limitations of our study, it is important to mention the small sample size of HS and control groups. However, such sample size was homogeneous enough to suggest associations between imaging (T2 and DWI) and neuropathological WM changes. Moreover, further validation of the present histological results could be performed in larger series of surgical cases, including etiologies other than HS. Finally, it could be considered that the influence of fixation in the immunoreactivity of the different types of tissues (surgical and autopsy specimens) in our investigation, an intrinsic limitation of studies in which autopsy samples are used as controls.

In conclusion, after performing histopathological and MRI analyses in the WM of the anterior temporal pole from patients with TLE-HS, we showed changes in cell constitution and myelin remodeling. As we evaluated tissue from individuals with chronic epilepsy, it is possible that the current set of histopathological and quantitative MRI changes is associated with plasticity of neuronal and glial cells of the TLE network. Future studies based on the present pathology-imaging approach may contribute with insights into tissue alterations correlating with MRI signal changes and into pathophysiological mechanisms of other structural lesions causing focal epilepsy.

## Data Availability Statement

The raw data supporting the conclusions of this article will be made available by the authors, without undue reservation.

## Ethics Statement

The studies involving human participants were reviewed and approved by Research Ethics Committee of the University of Campinas. The patients/participants provided their written informed consent to participate in this study.

## Author Contributions

BZ, IC, BC, LS, CY, FC, and FR: contributed to study design. BZ, IC, BC, LS, VC, KS, BA, MA, HT, CY, EG, FC, and FR: obtained and analyzed the data. BZ, IC, LS, VC, KS, FC, and FR: drafted the manuscript and figures. All authors contributed to the article and approved the submitted version.

## Funding

This study was sponsored by grants from the São Paulo Research Foundation (FAPESP; 2013/07559-3, 2019/08259-0, 2020/12651-0) and FAEPEX UNICAMP (2037/19).

## Conflict of Interest

The authors declare that the research was conducted in the absence of any commercial or financial relationships that could be construed as a potential conflict of interest.

## Publisher's Note

All claims expressed in this article are solely those of the authors and do not necessarily represent those of their affiliated organizations, or those of the publisher, the editors and the reviewers. Any product that may be evaluated in this article, or claim that may be made by its manufacturer, is not guaranteed or endorsed by the publisher.
